# Supplementary material for: On identifying collective displacements in apo-proteins that reveal eventual binding pathways
Source: PLoS Comput Biol. 2019 Jan 15;15(1):e1006665. doi: 10.1371/journal.pcbi.1006665 (PMC6333327; doi:10.1371/journal.pcbi.1006665)
Supplement: S1 Text — (PDF) [file pcbi.1006665.s001.pdf]

Supporting information for "On identifying collective displacements in apo- proteins that reveal eventual binding pathways"

Dube Dheeraj Prakashchand,† Navjeet Ahalawat,† Himanshu Khandelia,‡ Jagannath Mondal,*,† and Surajit Sengupta*,†

†*Tata Institute of Fundamental Research, Center for Interdisciplinary sciences, Hyderabad 500107, India*

‡*MEMPHYS, Center for Biomembrane Physics, Department of Physics, Chemistry and Pharmacy, University of Southern Denmark, 5230 Odense M, Denmark*

E-mail: jmondal@tifrh.res.in,+914020203091; surajit@tifrh.res.in,+914020203089

1. NAP: the projection formalism and some definitions

The concept of non-affine displacements have been thoroughly developed for periodic crystals in the work of Ganguly et al. (see Refs. 25, 26 and 27 of the main text). This is demonstrated in Fig.A for a two dimensional crystal. While for an affine displacement there is a single, homogeneous linear transformation, which takes us to the final displaced coordinates from the initial reference, coordinates, non-affine displacements can never be realised by such a linear transformation of initial coordinates. In the Fig.A, we show an affine shear transformation, while the non-affine one is a resultant of a shear plus an arbitrary displacement of particle 1. Such a resultant transformation can never be realised by a single linear transformation of the initial coordinates. We define non-affinity as the least squared error incurred by

representing such an arbitrary transformation by a "best fit" linear affine transformation over the local neighbourhood of any particular atom. An identical definition of course exists in three dimensions.

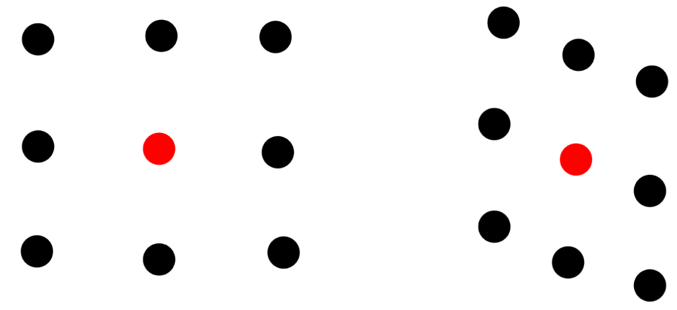

(a) Affine transformation (shear)

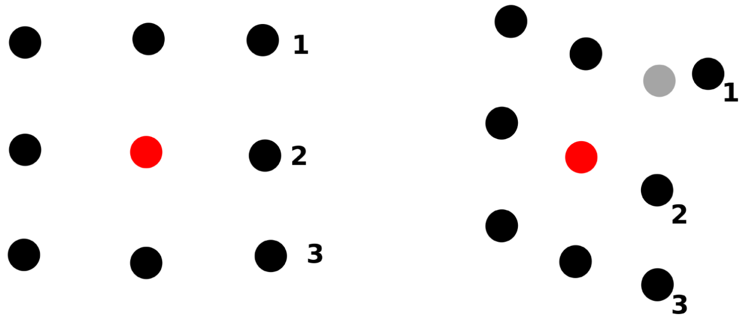

(b) Non-Affine transformation

Fig A. The affine transformation is actually a shear transformation, while the non-affine one is a resultant of shear plus displacement of particle 1. Such a resultant transformation can never be realized by a single linear transformation of the initial coordinates.

In order to adapt this formalism for proteins, two main modifications are necessary. Firstly, in a crystal, the reference configuration is the ideal lattice position of the atoms. In a protein, this is not so straightforward. We have taken as reference the native or average structure of the protein to calculate displacements. The reference coordinates of each atom

i is thus $\mathbf{R}_i$ while the instantaneous position is $\mathbf{r}_i$. The displacement, $\mathbf{u}_i = \mathbf{r}_i - \mathbf{R}_i$.

Secondly, in an ideal crystal with a single atom basis, the neighborhood of every atom is identical. For crystals with a basis, the number of unique neighborhoods is equal to the number of atoms in the basis. In a protein, on the other hand, the structural environment of every atom is unique. We have therefore fixed a "coarse graining radius" R_Ω which is used to define the neighborhood Ω_i around every atom. Note that all neighboring atoms lying within a sphere of radius R_Ω around the central atom i belongs to Ω_i. The number of atoms n_Ω within Ω_i neighborhoods of each atom in a protein therefore varies from atom to atom. The quantity R_Ω is a parameter of our method, which, in principle needs to be chosen with care. We discuss how R_Ω influences our results later.

Once R_Ω and the reference coordinates for every atom is fixed, the calculation proceeds as follows. The non-affine parameter, or local NAP value is given by,

$$\mathrm{NAP} = \min_{\mathcal{D}} \Big(\sum_{j=1}^{n_\Omega} [\mathbf{r}_j - \mathbf{r}_i - \mathcal{D}(\mathbf{R}_j - \mathbf{R}_i)]^2 \Big), \tag{1}$$

where the 3×3 dimensional deformation matrix $\mathcal{D} = \mathsf{I} + \varepsilon$, I the identity matrix and ε the sum of local strain and rotation. Writing the above equation in component form gives us,

$$\mathrm{NAP} = \min_{\varepsilon_{\mu\nu}} \sum_{j=1}^{n\Omega} \sum_{\mu=x,y,z} [(r^{\mu}{}_j - r^{\mu}{}_i) - \sum_{\nu=x,y,z} (\delta_{\mu\nu} + \varepsilon_{\mu\nu})(R^{\nu}{}_j - R^{\nu}{}_i)]^2 \tag{2}$$

The minimization over the components of ε for any particle i can be carried out in a straight forward manner leading to the following result.

$$\begin{aligned} \varepsilon^*_{\mu\nu} &= \sum_k [\mathrm{X}_{\mu\alpha} \mathrm{Y}^{-1}_{\nu\alpha}] - \delta_{\mu\nu} \qquad (3) \\ \mathrm{Y}_{\mu\nu} &= \sum_{j=1}^{n\Omega} [(R_j{}^{\mu} - R_i{}^{\mu})(R_j{}^{\nu} - R_i{}^{\nu})] \\ \mathrm{X}_{\mu\nu} &= \sum_{j=1}^{n_\Omega} [(r_j{}^{\mu} - r_i{}^{\mu})(R_j{}^{\nu} - R_i{}^{\nu})] \end{aligned}$$

The NAP may now be obtained by substituting ε^* in Eq.2. This completes the calculation of NAP for any atom i. The NAP susceptibility is obtained by calculating the statistical fluctuation of this value. To obtain greater insight into the meaning of NAP, and the non-affine eigenmodes etc. one needs to reformulate the least squared minimization problem outlined above within a projection operator formalism. We discuss this procedure below.

We begin by defining the relative displacements $\boldsymbol{\Delta}_j = \mathbf{u}_j - \mathbf{u}_i$, where we have suppressed the index i for simplicity. Using a term by term expansion one can show that NAP $= \min_{\varepsilon} \mathsf{S}^{\mathrm{T}}\mathsf{S}$ where the $3n_\Omega \times 1$ column vector $\mathsf{S}(\varepsilon) = \Delta - \mathsf{R}\tilde{\varepsilon}$ i.e. ,

$$\mathsf{S} = \begin{pmatrix} \Delta_{1x} \\ \Delta_{1y} \\ \Delta_{1z} \\ . \\ . \\ . \\ \Delta_{n_\Omega x} \\ \Delta_{n_\Omega y} \\ \Delta_{n_\Omega z} \end{pmatrix} - \begin{pmatrix} (R_1-R_0)_x & (R_1-R_0)_y & (R_1-R_0)_z & 0 & 0 & 0 & 0 & 0 & 0 \\ 0 & 0 & 0 & (R_1-R_0)_x & (R_1-R_0)_y & (R_1-R_0)_z & 0 & 0 & 0 \\ 0 & 0 & 0 & 0 & 0 & 0 & (R_1-R_0)_x & (R_1-R_0)_y & (R_1-R_0)_z \\ . & . & . & . & . & . & . & . & . \\ . & . & . & . & . & . & . & . & . \\ . & . & . & . & . & . & . & . & . \\ (R_{n_\Omega}-R_0)_x & (R_{n_\Omega}-R_0)_y & (R_{n_\Omega}-R_0)_z & 0 & 0 & 0 & 0 & 0 & 0 \\ 0 & 0 & 0 & (R_{n_\Omega}-R_0)_x & (R_{n_\Omega}-R_0)_y & (R_{n_\Omega}-R_0)_z & 0 & 0 & 0 \\ 0 & 0 & 0 & 0 & 0 & 0 & (R_{n_\Omega}-R_0)_x & (R_{n_\Omega}-R_0)_y & (R_{n_\Omega}-R_0)_z \end{pmatrix} \begin{pmatrix} \varepsilon_{xx} \\ \varepsilon_{xy} \\ \varepsilon_{xz} \\ \varepsilon_{yx} \\ \varepsilon_{yy} \\ \varepsilon_{yz} \\ \varepsilon_{zx} \\ \varepsilon_{zy} \\ \varepsilon_{zz} \end{pmatrix}$$

Carrying out the minimization with respect to the column vector $\tilde{\varepsilon}$ we obtain $\tilde{\varepsilon}^* = \mathsf{Q}\Delta$, where $\mathsf{Q} = (\mathsf{R}^{\mathrm{T}}\mathsf{R})^{-1}\mathsf{R}^{\mathrm{T}}$. Substituting in the expression for NAP, we finally obtain the microscopic value of NAP at the atom i as, NAP $= (\mathsf{P}\Delta)^{\mathrm{T}}(\mathsf{P}\Delta) = \Delta^{\mathrm{T}}\mathsf{P}\Delta$ where $\mathsf{P} = \mathsf{I} - \mathsf{R}\mathsf{Q}$. Note that $\mathsf{P}^{\mathrm{T}} = \mathsf{P}$ i.e. P is symmetric. Similarly $\mathsf{P}^2 = \mathsf{P}$ i.e. P is also idempotent. The matrix P is therefore a projection operator which projects out the *non-affine* component of the relative displacements Δ. The ensemble average of NAP is therefore given by $\langle \mathrm{NAP} \rangle = \langle (\mathsf{P}\Delta)^{\mathrm{T}}(\mathsf{P}\Delta) \rangle = \mathrm{Tr}\ \mathsf{P}\langle \Delta^{\mathrm{T}}\Delta \rangle \mathsf{P} = \mathrm{Tr}\ \mathsf{PCP}$, where we have used C to denote the average local displacement correlator and Tr denotes the trace operation. The displacement correlator for the entire protein in Fourier space is of course the dynamical matrix. NAP therefore measures the local displacement correlations *projected onto the non-affine sub-space*.

The eigenmodes of the $3n_\Omega \times 3n_\Omega$ dimensional matrix C exhaust all possible fluctuations

of the n_Ω atoms within Ω_i. Out of these only 9 modes are affine. All the rest of the modes are non-affine. The matrix PCP therefore has 9 zero eigenvalues which span the null space of this matrix. The eigenmodes of PCP corresponding to non-trivial eigenvalues constitute the important non-affine fluctuations of the atoms within Ω_i. The softest eigenmode corresponding to the largest eigenvalue of PCP contributes the most to NAP and constitutes the most important non-affine mode. The NAP value is basically the absolute value of all the non-affine modes of displacement. These modes of displacements basically result in reconfiguration of the native or the reference structure or conformational transformations.

The "gap", Γ_i is the difference between the first (largest) and second (second-largest) eigenvalues. Fig 1b basically demonstrates that the distribution of eigenvalues is non-uniform and the largest eigenvalue is separated from the rest by a large gap Γ_i and also this gap is relatively more prominent for the active regions than for the inert regions of the protein. In Fig 1b, each set of eigenvalues are normalized by dividing by the maximum eigenvalue in the respective set to obtain the comparison between the gaps of different sets on equal footing.

The NAP susceptibility is a response function for NAP under the effect of local field to which the protein system is exposed, just like C_v or isochoric heat capacity is a response function for internal energy of a system exposed to temperature gradient and compressibility is a response function for volume of a closed under the effect of pressure gradient. Just like fluid with high compressibility (like gases) can be compressed easily compared to those with low compressibility (liquids), similarly those regions of the proteins which have high value for NAP susceptibility undergo huge non-affine transformations in their conformations compared those with low NAP susceptibility.

Similarly, NAP spatial correlation function measures the correlation between non-affine displacement fluctuations at two different locations.

2. Notes on sensitivity of simulation-length on NAP Analysis

We had previously performed an analysis of the effect of simulation length over the NAP analysis. The NAP susceptibility values for all the particles in 200ns long trajectory was found have trends similar to that in case of 400ns trajectory. This is also seen in the Fig.B. The time-step of the MD frames chosen for both the case was 10ps. So increasing the simulation length does not much change the results. But increasing this time gap with a proprotionate increase of simulation length would help us in getting a better averaging. This is because the MD frames that we would be taking into account would be comparatively less correlated to each other when we increase the time gap between them. In other words, the practice of increasing the time-gap between the snapshots should be adopted to ensure that the snapshots are less correlated to each other and better averaging is obtained.

3. Details of different Regions of Proteins studied in the NAP analyses

L99A T4 Lysozyme , PDB ID 3DMV

The following are the 5 regions of T4Lys with high NAP susceptibiity:

regions	residues	description
**1**	105	part of helix-5
**2**	102	part of helix-5
**3**	137	part of helix-8
**4**	162 163 164	—
**5**	18 66 68	—

The other residues having moderate values of NAP susceptibility which appear as green

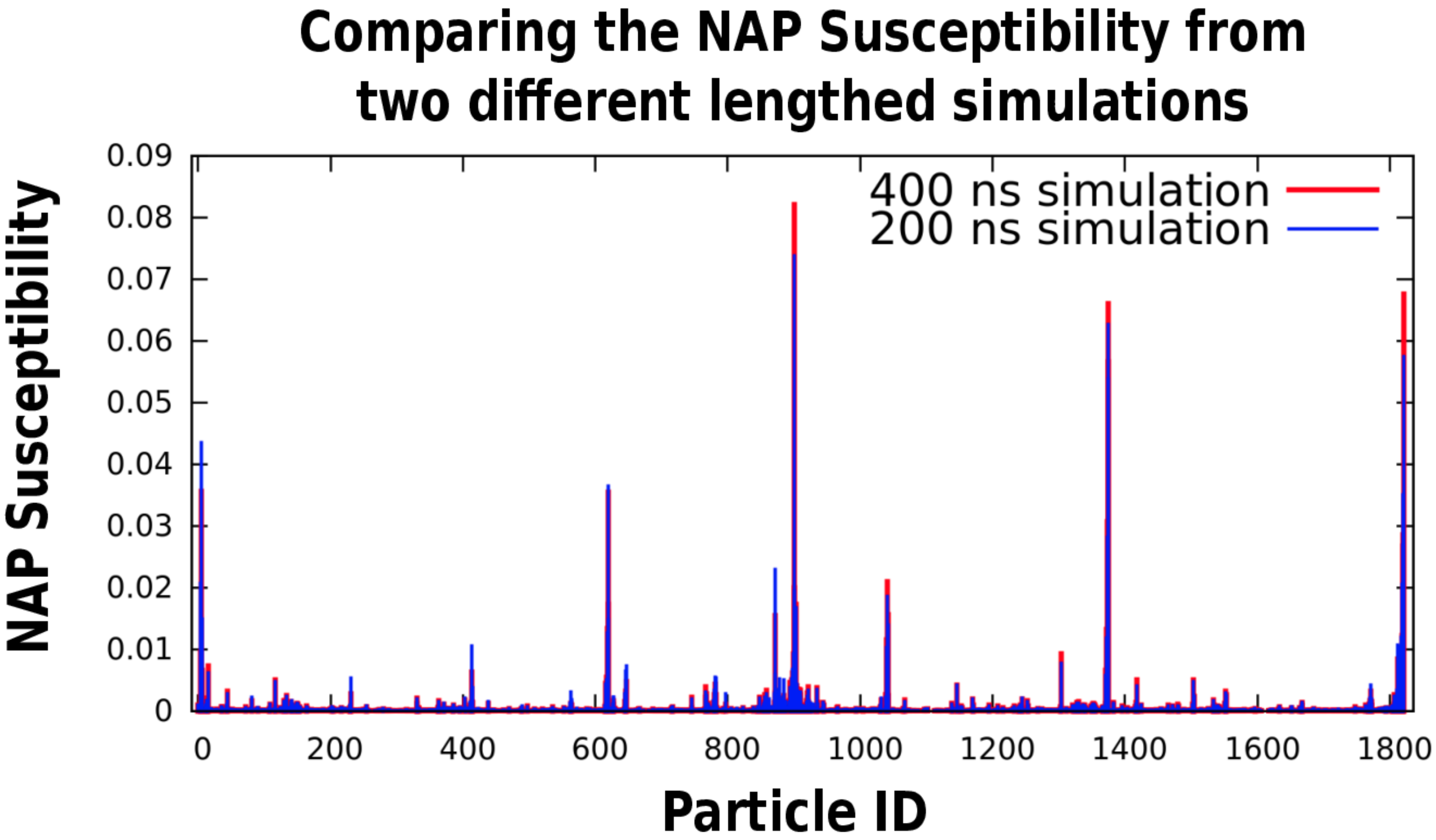

Fig B. The blue curve is the nap-susceptiblity values for the particles of Src-kinase calculated over 200ns and the red one is for 400ns. The regions of higher NAP susceptibility values captured are the same in both cases. For all our calculations, throughout our work, we choose to select conformations separated by 10ps time-gap.

spheres in figure 4a are : 133 ,76 ,145 ,11 ,21 ,106 ,20 ,108 ,29 ,122 ,9 ,104.

The relative values of the spatial NAP-correlation for the 5 locations shown in Fig.4(a) for T4Lys are as follows:

regions	residues	Correlation function value
**1,5**	18,105	0.55
**3,4**	133,164	0.67
**1,4**	105,164	0.71
**4,5**	18,164	0.75
**3,5**	18,137	1.00

Src kinase, PDB ID 1Y57

The following are the 5 regions of Src-kinase with large NAP susceptibility

regions	residues	description
**1**	310 311 315 329 328	PIF site
**2**	326 337 340 389	ATP binding site
**3**	481 482	G-loop site
**4**	360 364 457 484 486 487 488 489 525 526 532 533	MYR site
**5**	386 387 408 410 416 417 419 420 422 423 424 440	Activation loop region

The other residues having moderate values of NAP susceptibility which appear as green spheres in figure 4c are : 459 ,486.

The relative values of the spatial NAP-correlation for the 5 points shown in Fig.4(b) for Src-Kinase are as follows:

regions	residues	Correlation function value
**3,4**	481,486	0.25
**2,4**	326,532	0.35
**1,3**	481,310	0.39
**4,5**	533,416	0.87
**1,5**	328,416	1.00

4. Description of Videos

We have included four videos which show prominent non-affine displacements. In each of these videos we have applied the eigenvectors corresponding to the largest eigenvalue of the local PCP matrix to cause displacements of the atoms corresponding to the native structure of the protein. Note that these are not MD simulations.

NAME	Phenomenon
**S1**	opening-up of helices 4 and 6
**S2**	opening-up of helices 7 and 9
**S3**	close to open configuration
**S4**	allosteric contact: activation loop and Helix-αc

1. The video S1 demonstrates the opening of the helices 4 and 6 in order to enable the ligand to enter the binding site using the pathway depicted in Fig. 2 **a** of the manuscript. Note the twisting of the helices in this video and in V2 below.

2. The video S2 demonstrates the opening of the helices 7 and 9 in order to enable the ligand to enter the binding site using the pathway depicted in Fig. 2 **b** of the manuscript.

3. The video S3 demonstrates the non-affine modes for the residues having high spatial NAP correlation shown in Fig.4(a), thereby demonstrating the allosteric contact between regions 3 and 5 of T4Lys. The color scheme used is same as Fig.4(a). The loop in vicinity of region 3 of which ARG137 is a part is shown in black . and the loop of which TYR18 is a part, being a neighbourhood of region 5, is shown in yellow. The Alfa-carbon atoms of the two residues (TYR18 , ARG137 of regions 5 and 3 respectively) having high spatial NAP correlation are shown in pink solid bubbles. Here we see the residue 22(GLU) yellow, initially forming a hydrogen bond with residue ARG(137) black moving apart from residue 137 black. Thus, the non-affine modes at residue 18 and 137 act as nucleating precursors which tend to take T4Lys molecule from the closed conformation to open conformation.

4. The video S4 demonstrates the non-affine modes for the residues having high correlation shown in Fig.4(b), thereby demonstrating the allosteric contact between regions 1 and 5 of SRC-Kinase. The color scheme used is same as Fig.4(b). The activation loop alongwith residue Tyr416 being a part of region 5 is shown in yellow. and the Alfa-c

helix alongwith residue 328 being a neighbourhood of region 1 is shown in blue. The Alfa-carbon atoms of the two residues (Tyr416 , Val328 of regions 5 and 1 respectively) having high spatial NAP correlation are shown in pink solid bubbles. Here we see the residue 416 protruding outside from the activation loop region thereby becoming more exposed and facilitating the phosphorylation. At the same time, the Alfa-c helix is seen to be undergoing an inward rotation. Non-affine modes analysis show that the incipients for the outwards protruding of Tyr416 and incipients for the inwards rotation of Alfa-c helix have an allosteric contact. Apart from this, there is a slight distortion of the salt-bridge between 295LYS and 310GLU. The two residues 295LYS and 310GLU are shown in silver small spheres and stick form.

5. Notes on the sensitivity of choice of neighborhood-volume

In d-dimensional space, for a Coarse-graining volume (Ω_i) containing n_Ω number of particles , there are d^2 possible affine modes and the remaining $3N - d^2$ modes are non-affine. Therefore in 3 dimensions, out of the $3n_\Omega$ modes, 9 are naturally affine and the remaining $3n_\Omega - 9$ are non-affine. So, if there are only 3 particles inside Ω_i, the number of non-affine modes would be zero and hence the NAP value, which is the resultant magnitude of all the $3n_\Omega - 9$ non-affine modes after minimizing it, for the particle i would always be zero and that would never allow us to capture any non-affinity in the neighborhood of the particle i. So, the bare minimum number of particles for NAP calculation has to be 4. However dense regions of the protein involve many neighboring particles which can potentially influence the motion of the region. So, one needs to choose Ω_i judiciously. To show the effect of different coarse-graining radius (CGR) on the non-affinity of the protein, we have calculated the time profile of NAP values of helix7/helix9 of L99A T4 Lysozyme, by considering diverse range of CGR. This is plotted in Fig.C. We see that as the radius decreases, the NAP value becomes monotonically less

sensitive. This suggests that care needs to be exercised when choosing a Ω_i. In our article, we have chosen an intermediate value of CGR of 1.4 nm for all three systems, namely L99A T4 Lysozyme , Src-kinase and cytochrome-p450. The choice of CGR of 1.4 nm also can be rationalized by the fact that the distance between the helices varies approximately around 1.4nm (see fig 3 in main text). Any radius larger than this would no doubt capture non-affine signals in a similar trend as demonstrated by the green curve of Fig.C corresponding to radius 4.628 nm (this encloses entire molecule) where we see stronger signals compared to that obtained in red curve corresponding to the optimized radius of 1.4 nm. But the analysis of the non-affine modes for such large Ω_i would loose the local picture. For radii smaller than 1.4 nm, we see the signals diminishing. This is very clearly demonstrated in the Fig.C.

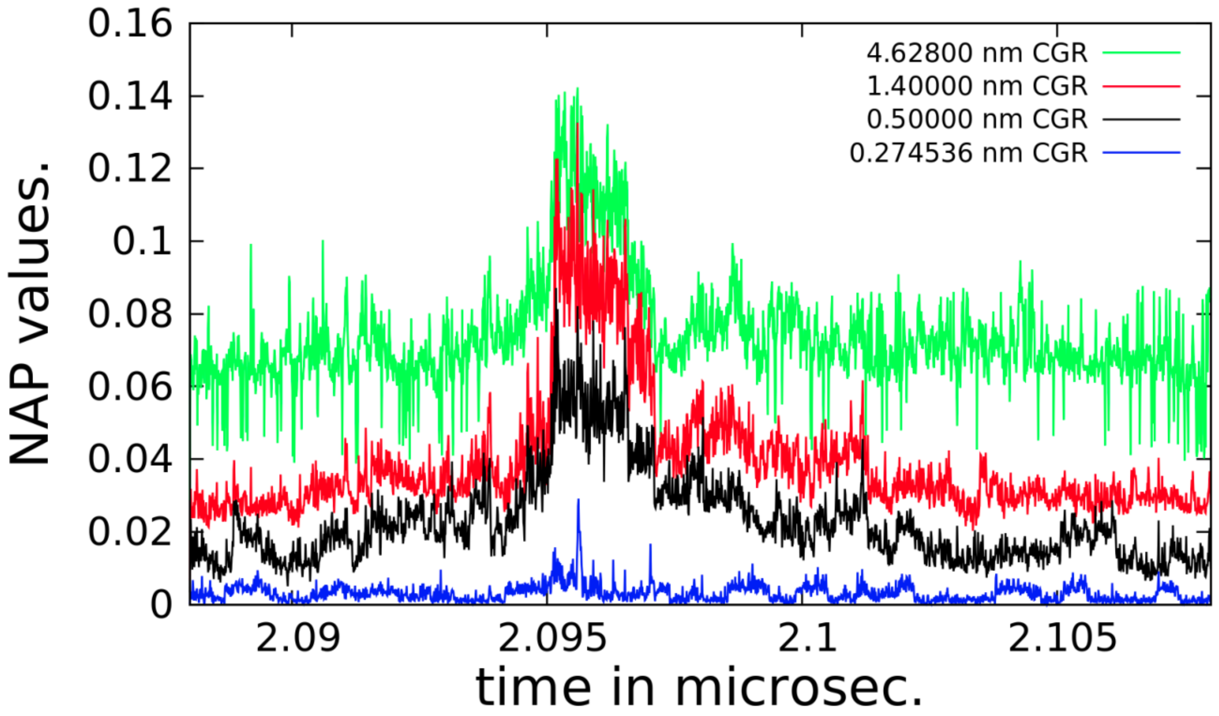

Fig C. This figure captures the time variation of NAP value (over the binding event for T4 lysozyme through the helices 7 and 9 acting as the gateways) calculated for the different Ω_i radii for C-alfa of residue 137ARG . For any future analyses, it could be suggested that the radius of Ω_i should be comparable to the length scale of secondary structure of prime focus. CGR in the key is Coarse-grained volume's radius.

6. Notes on the effect of choice of neighborhood on NAP eigen-spectrum

The figure Fig.D. demonstrates the effect of increasing the size of neighbourhood. Here, we observe that as the Coarse-graining radius (CGR) is increased the highest eigen value increases. Also the gap between the highest eigen value and the rest of the spectrum increases with increasing CGR, which is suggestive of an improvement in the resolution of non-affinity i.e. for larger CGR there emerges a very prominent non-affine eigen mode which governs the non-affine behaviour. For too small CGR, this high resolution is lost (there is no prominent non-affine mode obtained) and for too large CGR, as already stated in section - "Notes on sensitivity of choice of neighbourhood-volume", local picture is lost. Hence, it could be best suggested that the radius of Ω_i should be chosen to be comparable to the length scale of secondary structure of primary focus. This way we will have all the necessary local information contained in NAP values with good enough resolution.

7. Notes on sensitivity of main and side chains atoms on NAP Analysis

While analyzing the NAP values in a particular region of interest in protein (e.g. gateway regions helix 7-9 and helix 4-6 in L99A T4 Lysozyme binding to benzene), we take an average of the NAP values over all the particles from that region. There is no significant difference that we can see in our NAP analysis whether the side chain atoms are included in the averaging process or not. The Fig.E demonstrates this clearly. Through NAP analysis we record the behavior of any precursors of major conformational changes and according to our hypothesis such precursors for major conformational changes can nucleate due to any non-affine behavior shown by either side chain atoms or main chain atoms. Hence even the side-chain atoms' non-affine behavior becomes important and should be considered in the

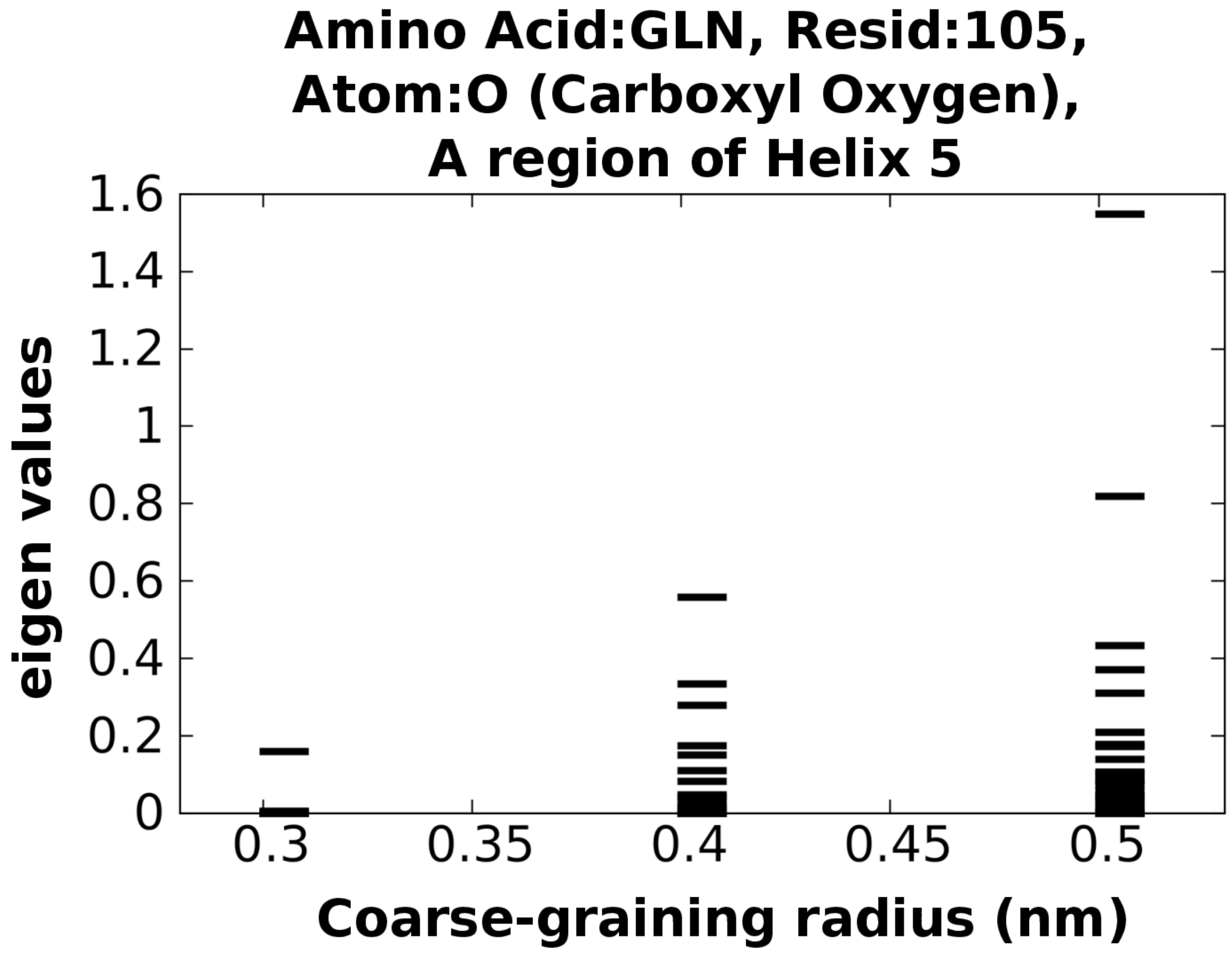

Fig D. In the above figure the eigen spectrum for three different radii have been plotted : 0.3nm , 0.4nm , 0.5nm for the residue GLN 105. This residue has the highest NAP susceptibility in the helix 5. The helix 5 acts as hinge region for the major non-affine transformations required during the binding through the helix-4-and-6 gateway.

averaging process. We wanted to take into account the statistics of all particles in calculating the NAP, rather than selectively choosing backbone or side-chain. Also, such an averaging practice actually helps us in bypassing the non-uniform behavior of side chain and main-chain atoms in displaying the non-affine signals or displacements. The same averaging applies for the study of allostery and susceptibility calculations.

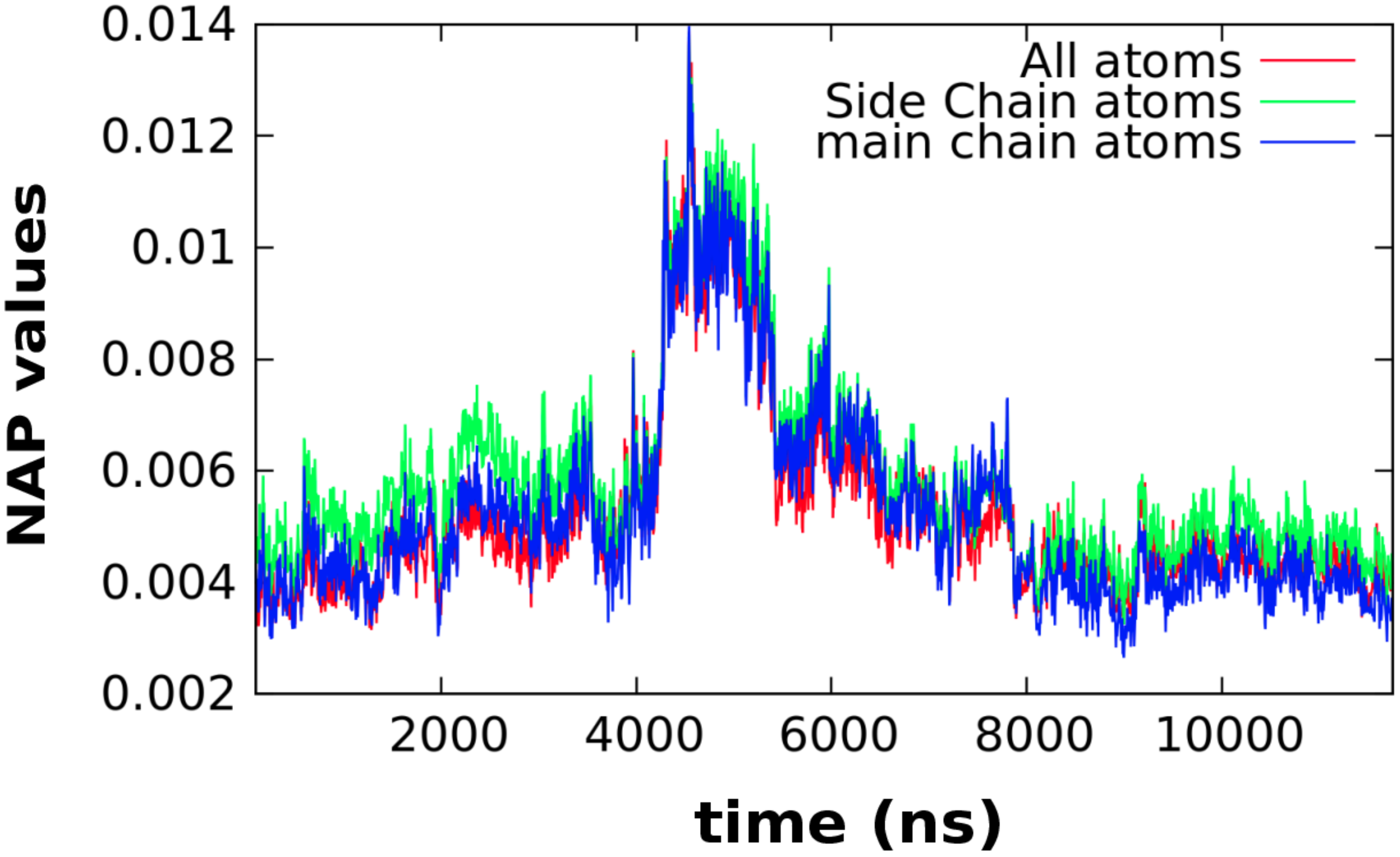

Fig E. The average value of NAP for the Side chain , for the main chain and for all the particles taken together almost show the same trend and same magnitude as demonstrated by the above figure. The calculation has been done for the region helix 7-9 of L99A T4 lysozyme while binding to benzene.

8. Relation with PCA

In our work non-affine modes of fluctuations are projections from the total displacements $\boldsymbol{\Delta}$ by allowing the operator or matrix P to act on $\boldsymbol{\Delta}$ i.e. we calculate $\mathsf{P}\boldsymbol{\Delta}$. We therefore perform a normal mode analysis over this projected subspace containing solely the non-affine modes of deformation by calculation of the local, projected Sample Correlation Matrix (SCM) $\langle(\mathsf{P}\boldsymbol{\Delta})^{\mathrm{T}}(\mathsf{P}\boldsymbol{\Delta})\rangle$ where angular brackets represent ensemble average for the trajectories of atoms in the ligand free protein. Carrying out this ensemble average we obtain the correlator as PCP. Here $\mathsf{C} = \langle\boldsymbol{\Delta}^{\mathrm{T}}\boldsymbol{\Delta}\rangle$ is an SCM for ordinary (total) displacement $\boldsymbol{\Delta}$ undergone by the

atoms within the coarse-grain volume.

We have shown that from a normal mode analysis of $\mathsf{P}\boldsymbol{\Delta}$ we obtain the eigenvalues and eigenvectors describing the most important and relevant dynamical modes for protein function. The softest eigenmodes or the eigenvectors corresponding to the largest eigenvalues are demonstrated in the videos provided in the supporting material (videos V1-V4). The modes of fluctuations shown in these videos indeed demonstrate that these non-affine modes are actually responsible for bringing about the important functions like the opening-up of the gateways leading to the binding pockets and the allosteric interactions between the distal functional sites.

This projection to the non-affine subspace is crucial. An ordinary principal component analysis (PCA) keeps the full displacement correlator of the whole protein without the projection. In Fig.F we show the result of this calculation for T4Lys. Understandably, none of the relevant conformation changes related to gate opening can be seen. The displacements are overwhelmingly affine.

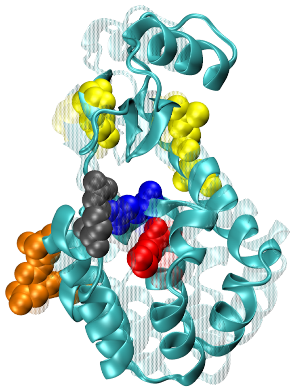

Fig F. The solid cyan image is the original crystal structure of T4 Lysozyme. The ghost cyan image is the final orientation of the protein after the application of the most prominent (highest eigenvalue) eigenmode obtained from the Principal Component Analysis. For comparison with our NAP susceptibility calculations, the regions of large susceptibility are highlighted with the same color scheme as in Fig.5 of the manuscript. Note that now no helix twisting motion can be seen in region 1 and 2. The PCA displacements are seen to be mostly affine.
